# Prefermentation improves xylose utilization in simultaneous saccharification and co-fermentation of pretreated spruce

**DOI:** 10.1186/1754-6834-2-8

**Published:** 2009-04-08

**Authors:** Magnus Bertilsson, Kim Olofsson, Gunnar Lidén

**Affiliations:** 1 Department of Chemical Engineering, Lund University, Box 124, SE-221 00 Lund, Sweden

## Abstract

**Background:**

Simultaneous saccharification and fermentation (SSF) is a promising process option for ethanol production from lignocellulosic materials. However, both the overall ethanol yield and the final ethanol concentration in the fermentation broth must be high. Hence, almost complete conversion of both hexoses and pentoses must be achieved in SSF at a high solid content. A principal difficulty is to obtain an efficient pentose uptake in the presence of high glucose and inhibitor concentrations. Initial glucose present in pretreated spruce decreases the xylose utilization by yeast, due to competitive inhibition of sugar transport. In the current work, prefermentation was studied as a possible means to overcome the problem of competitive inhibition. The free hexoses, initially present in the slurry, were in these experiments fermented before adding the enzymes, thereby lowering the glucose concentration.

**Results:**

This work shows that a high degree of xylose conversion and high ethanol yields can be achieved in SSF of pretreated spruce with a xylose fermenting strain of *Saccharomyces cerevisiae *(TMB3400) at 7% and 10% water insoluble solids (WIS). Prefermentation and fed-batch operation, both separately and in combination, improved xylose utilization. Up to 77% xylose utilization and 85% of theoretical ethanol yield (based on total sugars), giving a final ethanol concentration of 45 g L^-1^, were obtained in fed-batch SSF at 10% WIS when prefermentation was applied.

**Conclusion:**

Clearly, the mode of fermentation has a high impact on the xylose conversion by yeast in SSF. Prefermentation enhances xylose uptake most likely because of the reduced transport inhibition, in both batch and fed-batch operation. The process significance of this will be even greater for xylose-rich feedstocks.

## Background

Simultaneous saccharification and fermentation (SSF) [1] has proved to be an interesting option for ethanol production from lignocellulosic materials [2]. Critical factors for the process economy are a high final ethanol concentration in the fermentation along with a high overall ethanol yield [3]. In order to increase the ethanol concentration, a high content of water insoluble solids (WIS) is needed. At higher WIS contents, however, the concentrations of inhibitors and hexoses (inhibiting xylose uptake) will be higher. In addition, mixing problems may occur. These effects will probably be less pronounced in fed-batch SSF compared with the simple batch mode.

High overall ethanol yield requires conversion of all the sugars present, that is, both hexoses and pentoses, which can be achieved by, for example, genetically engineered strains of *Saccharomyces cerevisiae*. Principally, genes encoding xylose isomerase (XI) from bacteria and fungi [4,5], or genes encoding xylose reductase (XR) and xylitol dehydrogenase (XDH) from fungi [6,7] can be introduced. A well-known problem in *S. cerevisiae *is that xylose transport into the cell is inhibited by glucose, since xylose and glucose compete for the same transport systems [8,9] and the affinity for xylose is approximately 200-fold lower than for glucose [10]. Therefore, the glucose concentration must be low in order to obtain efficient xylose uptake. Mannose, present in spruce hydrolyzate, is also known to inhibit xylose uptake. Due to the lower affinity for mannose this effect is, however, not as strong as with glucose [9]. In this work we only focus on glucose.

Nevertheless, it has been shown that glucose enhances xylose utilization at low but non-zero concentrations [11,12], which can be attributed to induction of transporter systems [11,13], induction of glycolytic enzymes [14] and improved co-factor generation [11]. The positive effects of glucose on xylose uptake make SSF an interesting process concept for co-fermentation. After pretreatment, hemicellulosic sugars, i.e. the pentoses (and also hexoses), are present as monomers while a large fraction of the glucose content is present as glucan fibres in the WIS. Thus, monomeric glucose is continuously released by the action of enzymes during the process, which could favour pentose utilization. The xylose-utilizing strain of *S. cerevisiae *used in this work, TMB3400 [15], has previously been used for SSF of lignocellulosic materials rich in xylose. Öhgren et al [16] improved the ethanol yield on pretreated corn stover at 10% WIS from 46% to 54% based on glucose and xylose, when Baker's yeast was replaced by TMB3400. In another study [17], it was found that the strain TMB3400 was to be preferred over the yeast *Pichia stipitis *for SSF of pretreated sugarcane bagasse. A xylose utilization of about 40% was reported at 7.5% WIS and an ethanol yield on glucose and xylose of 59%. Good results have also recently been obtained on wheat straw [18] where about 40% of the xylose was utilized at 9% WIS giving an ethanol yield on glucose and xylose of 71%. In the case of spruce, the overall ethanol yield can be estimated to increase by about 8% if the xylose fraction is completely utilized in the SSF [19]. However, xylose conversion in spruce is expected to be challenging, due to the high glucose to xylose ratio. Furthermore, due to the relatively severe pretreatment conditions, the fermentability is expected to be lower in the spruce hydrolyzate [20].

In the current work, co-fermentation of glucose and xylose from pretreated spruce was studied using the xylose-fermenting yeast TMB3400 in lab-scale SSF experiments. In order to enhance the xylose uptake, the concept of prefermentation, that is, the fermentation of initially available free glucose in the liquid before addition of enzymes, was tested. The idea was to reduce the competitive inhibition on xylose uptake by hexoses in this way. Not only batch but also fed-batch experiments were carried out. The principal advantages of fed-batch relate to the lower viscosity during the process and the gradual addition of inhibitors and hexoses.

## Methods

### Raw material and pretreatment

Wood chips from spruce, about 2 to 10 mm in size, were provided by a Swedish sawmill (Widtsköfle Sågverk AB). The wood chips were impregnated in closed plastic bags for 20 min with SO_2 _(2.5%_w/w _moisture). Subsequently, the impregnated chips were steam-pretreated batch-wise (1.5 kg chips per batch) at 210°C for 5 min in a 10 L reactor [21]. The pretreated material was then stored at 4°C. The composition of the slurry is shown in Table 1. The water-insoluble and liquid fractions were separated by pressing and vacuum filtering. The two fractions were analyzed using NREL (National Renewable Energy Laboratories) standard procedures [22,23]. The WIS content of the slurry, measured by weighing the washed and dried (105°C) fibres, was 16.6%. Before the pretreated material was used for cultivation or SSF, 10 M NaOH was added to reach pH 4.9.

**Table 1:** Composition of the pretreated spruce material

**Solids (% of WIS)**		**Liquid (g L^-1^)**	
Glucan	53.6	Glucose	35.9
Mannan	-	Mannose	35.2
Galactan	-	Galactose	6.3
Xylan	-	Xylose	11.6
Lignin	42.5	HMF	3.9
		Furfural	2.2
		Acetic acid	5.9

The WIS content was 16.6%.

### Cell cultivation

The recombinant xylose-fermenting strain *S. cerevisiae *TMB3400 [15] was used in the SSF experiments. Yeast cells to be used in SSF were produced by aerobic batch cultivation on glucose, followed by an aerobic fed-batch cultivation on spruce hydrolyzate liquid, in order to improve inhibitor tolerance by adaptation as previously shown by Alkasrawi et al [24].

The yeast was inoculated in 300 mL flasks containing 100 mL media supplemented with 16.5 g L^-1 ^glucose, 7.5 g L^-1 ^(NH_4_)_2_SO_4_, 3.5 g L^-1 ^KH_2_PO_4_, 0.74 g L^-1 ^MgSO_4_·7H_2_O, 1 mL trace metal solution and 0.1 mL vitamin solution. The trace metal and vitamin solutions were prepared according to Taherzadeh et al [25]. The cells were grown for 24 h at 30°C and pH 5 in a rotary shaker at 160 rpm.

Aerobic batch cultivation was performed in a 2.5 L bioreactor (Biostat A, B. Braun Biotech International, Melsungen, Germany) at 30°C. The working volume was 0.7 L and the medium contained 20.0 g L^-1 ^glucose, 20.0 g L^-1 ^(NH_4_)_2_SO_4_, 10.0 g L^-1 ^KH_2_PO_4_, 2.0 g L^-1 ^MgSO_4_, 27.0 mL L^-1 ^trace metal solution and 2.7 mL L^-1 ^vitamin solution. The cultivation was initiated by adding 20.0 mL of the inoculum to the bioreactor. Aeration was maintained at 1.0 L min^-1 ^and the stirrer speed was kept at 700 rpm. Upon depletion of the ethanol produced in the batch phase, the feeding of liquid from the pretreated material was initiated. 1.0 L of the liquid fraction was added with an initial feed rate of 0.04 L h^-1 ^that was increased linearly to 0.10 L h^-1 ^during 16 h of cultivation. The aeration during the fed-batch phase was maintained at 1.4 L min^-1 ^and the stirrer speed was kept at 700 rpm. The pH was maintained at 5.0 throughout the cultivation, by automatic addition of 3 M NaOH.

After cultivation the cells were harvested by centrifugation in 700 mL flasks using a HERMLE Z 513K centrifuge (HERMLE Labortechnik, Wehingen, Germany). The pellets were resuspended in 0.9% NaCl solution in order to obtain a cell suspension with a dry weight of 75 g L^-1^. The time between cell harvest and initiation of the SSF was no longer than 4 h.

### Simultaneous saccharification and fermentation

All SSF experiments were carried out in 2.5 L bioreactors (Biostat A, B. Braun Biotech International, Melsungen, Germany; Biostat A plus, Sartorius, Melsungen, Germany and BIOFLO III, New Brunswick Scientific, Edison, NJ, USA) with a final working weight of 1.4 kg. The batch experiments were carried out with a content of 7% or 10% WIS. The fed-batch experiments were performed with an initial WIS content of 6%, and slurry was added in equal pulses every fifth hour up to 20 h of fermentation. After the slurry additions, the total amount of slurry (initial and added amount) corresponded to a WIS content of 10%. All SSF experiments were carried out at 34°C for 96 h, and the pH was maintained at 5.0 throughout the fermentation by automatic addition of 3 M NaOH. To obtain the desired WIS content, the slurry was diluted with sterile deionized water. The SSF medium was supplemented to obtain 0.5 g L^-1 ^NH_4_H_2_PO_4_, 0.025 g L^-1 ^MgSO_4_·7H_2_O and 1.0 g L^-1 ^yeast extract. The experiments were initiated by addition of a dry weight concentration of 4 g L^-1 ^cells. The enzyme preparation, Celluclast, was provided by Novozymes A/S, Bagsvaerd, Denmark, and had a cellulase activity of 35 FPU g^-1 ^and a β-glucosidase activity of 20 IU g^-1^. This was used together with Novozyme 188 (Novozymes A/S, Bagsvaerd, Denmark) with a β-glucosidase activity of 339 IU g^-1^. The amount of enzyme added corresponded to a cellulase activity of 30 FPU g^-1 ^glucan, and a total β-glucosidase activity of 60 IU g^-1 ^glucan. Prefermentation was applied by adding the enzymes when glucose was almost depleted (<1 g L^-1^), i.e. at 7 h in experiments at 7% WIS and at 10 h and 3 h in batch and fed-batch experiments, respectively, at 10% WIS. Samples for high performance liquid chromatography (HPLC) analysis were taken repeatedly throughout the SSF.

### Analysis

The cell mass concentration during the cell cultivation was measured in duplicates from 10 mL samples. The samples were centrifuged at 1000 × g for 7 min (Z200 A, HERMLE Labortechnik, Wehingen, Germany). The supernatants were discarded, and the pellets were washed with 0.9% NaCl solution and centrifuged a second time. The pellets were dried at 105°C overnight and subsequently weighed.

HPLC was used for analysis of the metabolites and substrates. Samples of the fermentation liquid were centrifuged at 16,000 × g in 2 mL Eppendorf tubes for 5 min (Z 160 M, HERMLE Labortechnik, Wehingen, Germany). The supernatant was filtered using 0.2 μm sterile filters and the filtered samples were stored at -20°C. The sugar concentrations were determined using a polymer column (Aminex HPX-87P, Bio-Rad Laboratories, München, Germany) at 85°C. MilliQ-water was used as eluent, with a flow rate of 0.6 mL min^-1^. Ethanol, glycerol, acetate, HMF and furfural were analyzed using an Aminex HPX-87H column (Bio-Rad Laboratories, München, Germany) at 60°C. The eluent was 5 mM H_2_SO_4 _with a flow rate of 0.6 mL min^-1^. The compounds of interest were detected with a refractive index detector (Waters 2410, Waters, Milford, MA, USA).

### Yield calculations

The ethanol yield, Y_SE_, was calculated based on the total amount of fermentable sugars added to the SSF, i.e. the sum of available glucose, mannose, galactose and xylose present in the slurry, including both monomers and oligomers in the liquid and glucan fibres in the WIS. The theoretical weight of glucose released during the hydrolysis was (due to the addition of water) 1.11 times the weight of glucan. By using a maximal theoretical ethanol yield of 0.51 g g^-1 ^(yield for hexoses) the percentage of the theoretical yield was defined as Y*_SE _= 100*Y_SE_/0.51. When our results are related to previous results with Baker's yeast, the yields are given on available hexoses (glucose, mannose and galactose) only, in order to be comparable.

## Results

The performance of TMB3400, adapted by precultivation on spruce hydrolyzate, in SSF of pretreated spruce was studied in batch experiments at 7% WIS, and in both batch and fed-batch experiments at 10% WIS. Prefermentation was investigated as a means to decrease the glucose level and improve the xylose conversion (Table 2).

**Table 2:** Ethanol yields and concentrations of metabolites obtained after 96 h

**SSF mode**	**WIS**	**Prefermentation**	**Xylose consumption^1^** **%**	**Xylitol** **%^2 ^(g L^-1^)**	**Glycerol** **g L^-1^**	**Ethanol yield** **g g^-1^**	**Ethanol yield** **% of theoretical**	**Ethanol** **g L^-1^**
Batch	7%	No	77	27 (0.91)	2.9	0.44	86	32.0
Batch	7%	Yes	84	24 (0.86)	3.4	0.46	91	33.9
Batch	10%	No	16	27 (0.29)	3.6	0.38	75	39.6
Batch^3^	10%	Yes	31	24 (0.49)	3.6	0.41	81	43.0
Batch^3^	10%	Yes	42	23 (0.68)	4.8	0.41	80	42.3
Fed-batch	10%	No	56	24 (0.92)	3.9	0.43	85	45.0
Fed-batch	10%	Yes	77	22 (1.16)	4.2	0.43	85	45.2

^1^Related to total amount of available xylose.

^2^Xylitol formation related to consumed xylose.

^3^Batch experiment with prefermentation at 10% was run in duplicates.

### Batch SSF at 7% WIS

**Figure 1:**
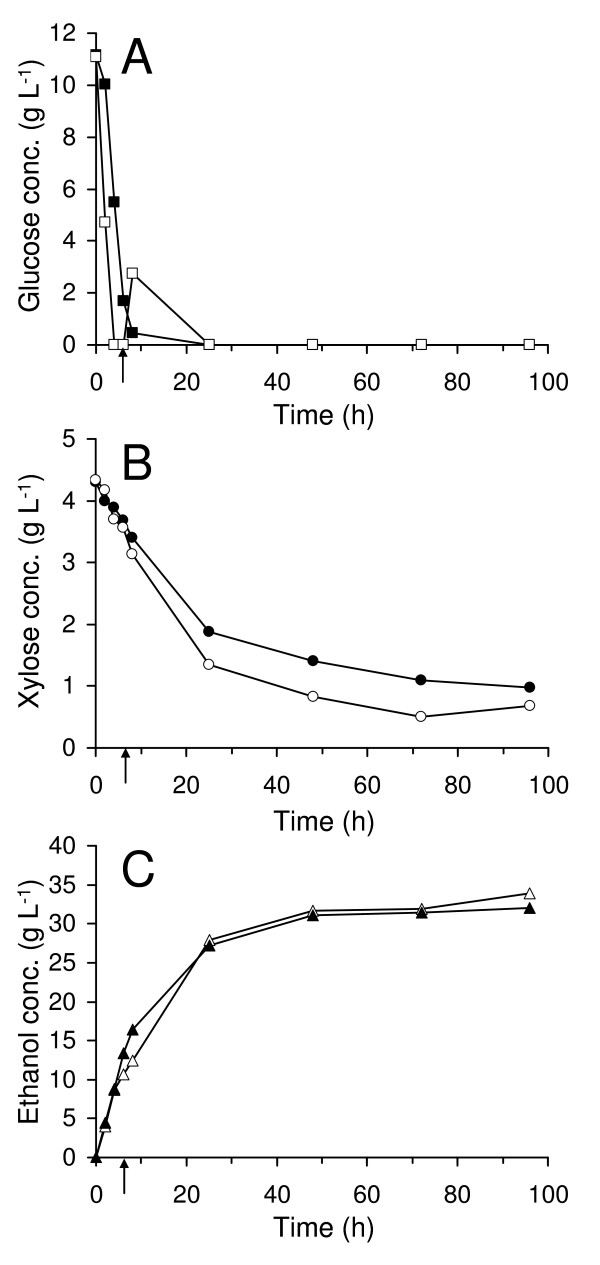
**Measured concentrations in batch SSF at 7% WIS**. Glucose concentration (A), xylose concentration (B) and ethanol concentration (C). Filled symbols represent experiment without prefermentation and unfilled symbols represent experiment with prefermentation. The time of enzyme addition, in the case of prefermentation, is marked with an arrow.

At 7% WIS, the glucose concentration was quickly reduced to 0 g L^-1 ^within 24 h. The glucose level was then maintained at this level throughout the SSF in both experiments (Figure 1A). After 96 h, 77% of the xylose was utilized without applying prefermentation. An ethanol concentration of 32.0 g L^-1 ^was achieved, corresponding to a yield of 0.44 g g^-1 ^on total sugars (86% of the theoretical yield). By applying prefermentation, a xylose utilization as high as 84% was achieved (Figure 1B). The difference in final xylose concentration was however low compared with the total sugar content.

### Batch SSF at 10% WIS

**Figure 2:**
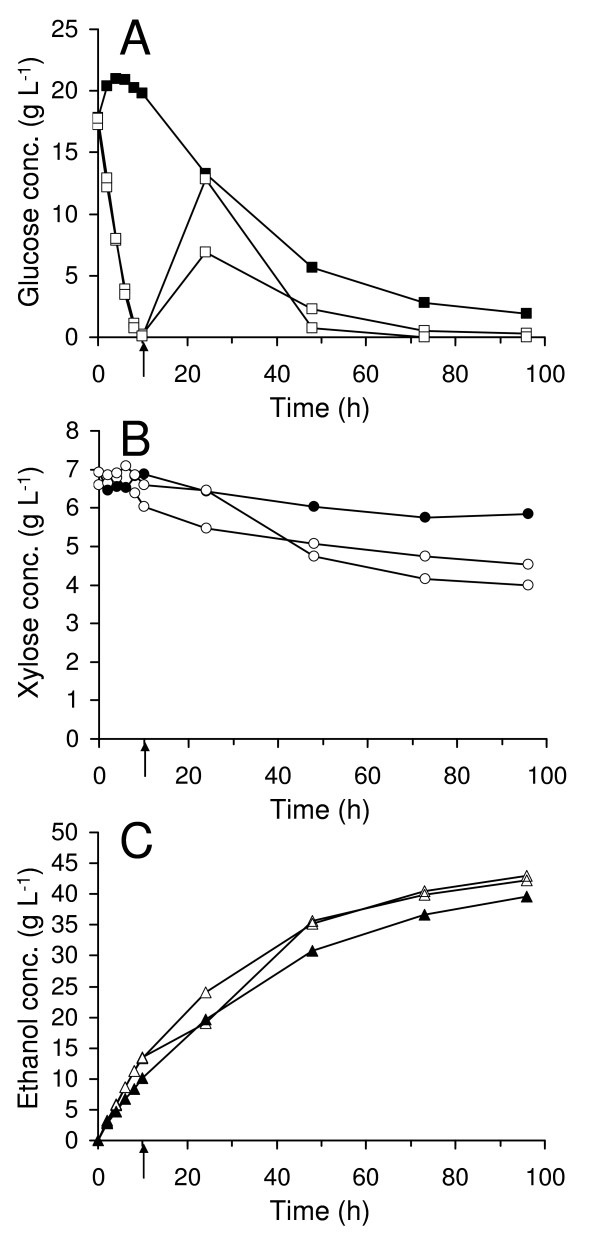
**Measured concentrations in batch SSF at 10% WIS**. Glucose concentration (A), xylose concentration (B) and ethanol concentration (C). Filled symbols represent experiment without prefermentation and unfilled symbols represent duplicate experiments with prefermentation. The time of enzyme addition, in the case of prefermentation, is marked with an arrow.

In a commercial bioethanol process, it is desirable that the substrate load is higher than 7% WIS. Experiments were conducted also at 10% WIS, but not at higher WIS content due to limitations of mixing and fermentability. As expected, the utilization of xylose was lower than at 7% WIS, and only 16% of the xylose was utilized in the experiment without prefermentation at 10% WIS. The final ethanol concentration reached 39.6 g L^-1 ^and the ethanol yield was 0.38 g g^-1 ^on total fermentable sugars, corresponding to 75% of the theoretical yield. Prefermentation at 10% WIS resulted as intended in lower glucose concentration throughout the fermentations (Figure 2A) accompanied by higher xylose conversions (Figure 2B). In these experiments (with prefermentation) 31% and 42% of the xylose was taken up respectively. This resulted in ethanol yields of about 0.41 g g^-1 ^on total fermentable sugars, or about 80% of the theoretical yield in both experiments (Table 2). The increased ethanol yield, when prefermenting the initial sugars, was partly due to the improved xylose utilization, but may also be partly explained by a slightly higher hexose conversion in these experiments. However, the measured final concentrations of sugars in these experiments do not completely explain the higher ethanol (and by-product formations) in the case of prefermentation, indicating that more glucose must have been released from the fibres in these experiments.

### Fed-batch SSF at 10% WIS

**Figure 3:**
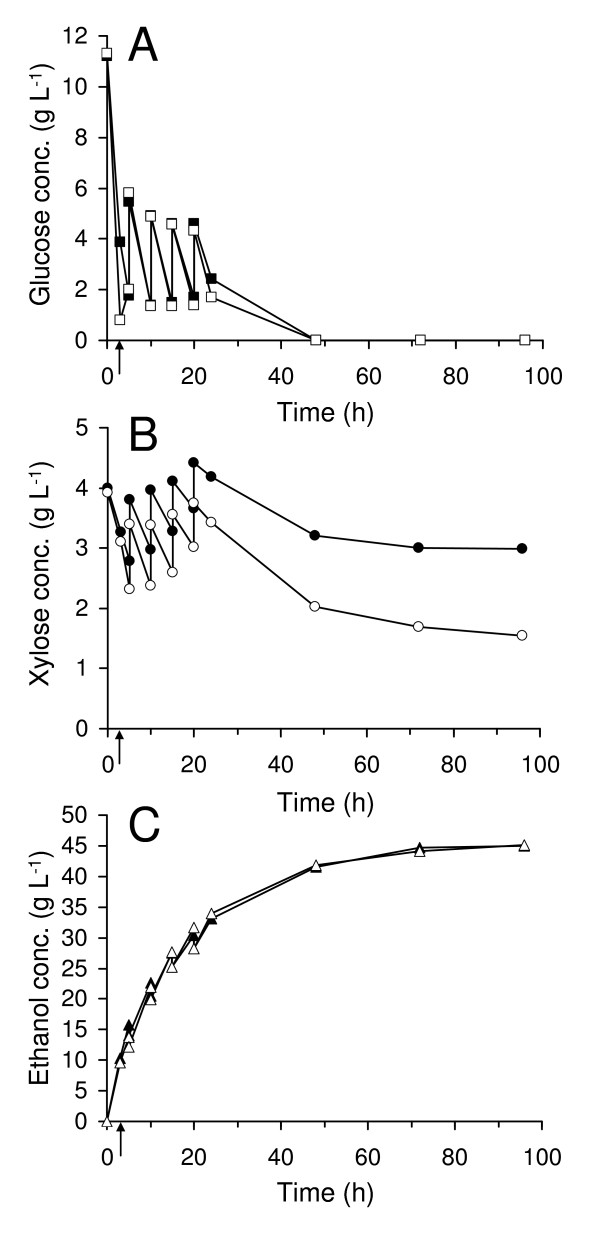
**Measured concentrations in fed-batch SSF at 10% WIS**. Glucose concentration (A), xylose concentration (B) and ethanol concentration (C). Filled symbols represent experiment without prefermentation and unfilled symbols represent experiment with prefermentation. The time of enzyme addition, in the case of prefermentation, is marked with an arrow.

The glucose concentration in the reactor can be further controlled by running the SSF in a fed-batch mode, that is, with the pretreated material added gradually, either continuously or (as in this case) at specific time points during the experiments. The fed-batch experiments resulted in higher xylose conversions than the batch experiments. Also in fed-batch operation, prefermentation seemed to improve the xylose consumption (Figure 3B) and 77% of the xylose was taken up. An ethanol yield of 85% of the theoretical yield was achieved.

## Discussion

Simultaneous co-fermentation and saccharification of spruce using the recombinant strain *S. cerevisiae *TMB3400 was experimentally investigated in the current study. Co-fermentation of xylose and glucose is a rather challenging task, given the high ratio of glucose to xylose in the spruce material itself. The concept of prefermentation, that is, the idea to initially convert the free glucose in the medium before adding enzymes, was therefore introduced in order to improve xylose uptake in SSF. This was also combined with fed-batch, which provides a further degree of freedom in terms of affecting the free glucose to xylose ratio.

In SSF at 7% WIS, the xylose was extensively taken up, both when the enzymes were added initially and when prefermentation was applied (Table 2), resulting in high ethanol yields. In these experiments, the competitive glucose inhibition of xylose uptake was reduced at an early stage, due to relatively low amount of initial glucose and inhibitors. Thus, in this case prefermentation becomes less important. In this experiment xylose uptake seemed to be boosted rather than inhibited by the small glucose overshoot that appeared after enzyme addition (Figure 1) in the case of prefermentation. Previous studies on spruce with Baker's yeast have resulted in 91% of theoretical ethanol yield on hexoses (glucose, mannose and galactose) at 5% WIS [26] and 92% at 8% WIS [24]. Due to extensive xylose utilization in this study, 97% of theoretical ethanol yield was achieved.

In batch SSF at 10% WIS, TMB3400 exhibited difficulties in completely fermenting glucose within 96 h in the ordinary batch experiment (Figure 2A). A residual concentration of about 2 g L^-1 ^was still found at the end of the experiment. The xylose uptake, in percent, at this high WIS concentration was much lower compared with when 7% WIS was used. The main reason is probably the large amount of glucose available. By increasing the WIS content, the concentrations of various inhibitors from the pretreatment are also increased, which most likely also lowers the fermentability. The batch experiment at 10% WIS with prefermentation was run in duplicate (Figure 2) to allow for estimation of variances. The standard deviation of the final xylose uptake and ethanol concentration was estimated to be <20% and <2% of the respective means. These experiments also illustrate the difficulty of reproducing the exact glucose profile from the time of enzyme addition when carrying out prefermentation experiments. This can possibly be due to the glucose-sensing mechanisms in yeast (for example, induction of hexose transporters) [27]. When glucose concentration approaches zero, small differences in concentration at the time of enzyme addition, may highly influence the uptake rate, giving rise to different degrees of glucose accumulation.

However, at 10% WIS, prefermentation had a considerable effect, resulting in lower glucose levels which significantly enhanced the xylose consumption from 16% to 31% and 42%, respectively. In addition, slightly more glucose seemed to be released from the fibres. Thus, prefermentation possesses another possible advantage – it may reduce the end-product inhibition of cellulases, allowing more efficient hydrolysis. These two effects together probably account for the higher ethanol yield in the case of prefermentation.

An alternative explanation may be the mechanisms of sugar transporter inactivation. Non-growing cells exhibit inactivation of the sugar transporters [28] in the presence of a fermentable carbon source. However, if no carbon source is available, catabolite inactivation does not occur [29], or occurs only marginally [30]. There are previous indications that no growth occurs in SSF of spruce at high WIS content [31]. The rapid hexose depletion in the case of prefermentation could therefore reduce the effect of catabolite inactivation. In contrast, when prefermentation is not applied, catabolite inactivation of sugar transporters may be a reason for lower xylose uptake and incomplete hexose fermentation.

Batch experiments on other spruce materials have indicated that in harsher environments, prefermentation does not always improve the overall SSF. However, xylose uptake is consistently enhanced by prefermentation during the initial 24 h also in these materials.

Previously, Rudolf et al [31] carried out SSF of spruce at 10% WIS using Baker's yeast, and ethanol yields of 80 to 84% on hexoses for batch SSF were reported. In this study, corresponding batch yields were 79 to 86%. Thus, despite partial xylose utilization, SSF of spruce with TMB3400 did not necessarily improve the ethanol yields in batch experiments at 10% WIS. The relatively low xylose content in spruce, and by-product formation, may explain this absence of improvement in batch. TMB3400 has been shown to produce more glycerol than Baker's yeast, which reduces the ethanol formed from xylose [32].

In agreement with previous SSF studies with TMB3400 using other feedstocks [16,18], the fed-batch strategy yielded the highest xylose uptakes at 10% WIS. This can be principally explained by the lower glucose levels, and the lower concentrations of toxic compounds. In the present study, 77% xylose conversion and as high yields as 85% of theoretical on total sugars (90% calculated based on hexoses) or 45.2 g L^-1 ^ethanol were achieved within 96 h when combining fed-batch and prefermentation. Yields of 78 to 84% on hexoses have previously been reported from similar fed-batch experiments with Baker's yeast [31]. Thus, when using the fed-batch strategy TMB3400 gives higher yields than ordinary Baker's yeast. Prefermentation improved xylose transport, also in these experiments, although this extra xylose consumption led mostly to by-products. Hence, there is a need for further engineering of the yeast in order to effectively utilize the xylose that is taken up.

Interestingly, not much xylose was taken up after 48 h in any of the SSF experiments, despite the fact that the concentration of hexoses was generally very low and xylose was available in significant amounts. This behaviour has also been seen in previous SSF studies with TMB3400 [16-18] and can probably be attributed to decreasing viabilities with time [31] in combination with very low rates of hydrolysis and possibly inactivation of transport systems as discussed above. Therefore, it is important to possess good xylose conversion conditions in the reactor at an early stage of the SSF. In previous SSF studies with TMB3400, the process has not been optimized for glucose and xylose co-fermentation. This work introduces prefermentation to rapidly reduce the initial glucose concentration and thereby promote good xylose uptake conditions at an early stage of the SSF. The process can potentially be further improved to achieve optimal co-fermentation conditions by balancing the formation rate (by the action of enzymes) and consumption rate of glucose (by fermentation). This involves optimizing the enzyme addition, the feed of enzymes, the feed of substrate – and perhaps even a feed of yeast. In addition, the possible effects of mannose on the xylose uptake should also be considered in future work on mannose-rich materials.

## Conclusion

This work shows that although pretreated spruce contains high levels of glucose and considerably less xylose, a high degree of xylose conversion can be obtained with the xylose-fermenting yeast strain TMB3400 in SSF at high WIS contents. Thereby higher ethanol yields than with ordinary Baker's yeast are possible. Furthermore, by prefermentation of initial hexoses, xylose uptake can be improved in SSF. The main benefits of prefermentation are, however, expected in other more xylose-rich materials.

## Competing interests

The authors declare that they have no competing interests.

## Authors' contributions

MB carried out the major part of the experimental work and made a first draft. KO carried out part of the experimental work. GL initiated the study, set the overall goals of the work and contributed to the design of the study. All authors were actively contributing to the discussion, during the whole work, and the preparation of the final manuscript. All authors have read and approved the final manuscript.

## References

[B1] Takagi M, Abe S, Suzuki S, Emert GH, Yata N (1977). A method for production of alcohol directly from cellulose using cellulase and yeast. Bioconversion Symposium; New Dehli, India.

[B2] Wingren A, Galbe M, Zacchi G (2003). Techno-economic evaluation of producing ethanol from softwood: Comparison of SSF and SHF and identification of bottlenecks. Biotechnol Prog.

[B3] von Sivers M, Zacchi G (1996). Ethanol from lignocellulosics: a review of the economy. Bioresour Technol.

[B4] Karhumaa K, Hahn-Hägerdal B, Gorwa-Grauslund MF (2005). Investigation of limiting metabolic steps in the utilization of xylose by recombinant *Saccharomyces cerevisiae *using metabolic engineering. Yeast.

[B5] Kuyper M, Hartog MMP, Toirkens MJ, Almering MJH, Winkler AA, van Dijken JP, Pronk JT (2005). Metabolic engineering of a xylose-isomerase-expressing *Saccharomyces cerevisiae *strain for rapid anaerobic xylose fermentation. FEMS Yeast Res.

[B6] Kötter P, Amore R, Hollenberg CP, Ciriacy M (1990). Isolation and characterization of the *Pichia stipitis *xylitol dehydrogenase gene, XYL2, and construction of a xylose-utilizing *Saccharomyces cerevisiae *transformant. Curr Genet.

[B7] Eliasson A, Christensson C, Wahlbom CF, Hahn-Hägerdal B (2000). Anaerobic xylose fermentation by recombinant *Saccharomyces cerevisiae *carrying XYL1, XYL2, and XKS1 in mineral medium chemostat cultures. Appl Environ Microbiol.

[B8] Kilian SG, Uden N (1988). Transport of xylose and glucose in the xylose-fermenting yeast *Pichia stipitis*. Appl Microbiol Biotechnol.

[B9] Meinander NQ, Hahn-Hägerdal B (1997). Influence of cosubstrate concentration on xylose conversion by recombinant, XYL1-expressing *Saccharomyces cerevisiae*: a comparison of different sugars and ethanol as cosubstrates. Appl Environ Microbiol.

[B10] Kötter P, Ciriacy M (1993). Xylose fermentation by *Saccharomyces cerevisiae*. Appl Microbiol Biotechnol.

[B11] Pitkänen J-P, Aristidou A, Salusjärvi L, Ruohonen L, Penttilä M (2003). Metabolic flux analysis of xylose metabolism in recombinant *Saccharomyces cerevisiae *using continuous culture. Metab Eng.

[B12] Meinander NQ, Boels I, Hahn-Hägerdal B (1999). Fermentation of xylose/glucose mixtures by metabolically engineered *Saccharomyces cerevisiae *strains expressing XYL1 and XYL2 from *Pichia stipitis *with and without overexpression of TAL1. Bioresour Technol.

[B13] Bertilsson M, Andersson J, Lidén G (2008). Modeling simultaneous glucose and xylose uptake in *Saccharomyces cerevisiae *from kinetics and gene expression of sugar transporters. Bioprocess Biosyst Eng.

[B14] Boles E, Müller S, Zimmermann FK (1996). A multi-layered sensory system controls yeast glycolytic gene expression. Mol Microbiol.

[B15] Wahlbom CF, van Zyl WH, Jonsson LJ, Hahn-Hägerdal B, Otero RRC (2003). Generation of the improved recombinant xylose-utilizing *Saccharomyces cerevisiae *TMB 3400 by random mutagenesis and physiological comparison with *Pichia stipitis *CBS 6054. FEMS Yeast Res.

[B16] Öhgren K, Bengtsson O, Gorwa-Grauslund MF, Galbe M, Hahn-Hägerdal B, Zacchi G (2006). Simultaneous saccharification and co-fermentation of glucose and xylose in steam-pretreated corn stover at high fiber content with *Saccharomyces cerevisiae *TMB3400. J Biotechnol.

[B17] Rudolf A, Baudel H, Zacchi G, Hahn-Hägerdal B, Lidén G (2008). Simultaneous saccharification and fermentation of steam-pretreated bagasse using *Saccharomyces cerevisiae *TMB3400 and *Pichia stipitis *CBS6054. Biotechnol Bioeng.

[B18] Olofsson K, Rudolf A, Lidén G (2008). Designing simultaneous saccharification and fermentation for improved xylose conversion by a recombinant strain of *Saccharomyces cerevisiae*. J Biotechnol.

[B19] Sassner P, Galbe M, Zacchi G (2008). Techno-economic evaluation of bioethanol production from three different lignocellulosic materials. Biomass Bioenerg.

[B20] Palmqvist E, Hahn-Hägerdal B (2000). Fermentation of lignocellulosic hydrolysates. II: inhibitors and mechanisms of inhibition. Bioresour Technol.

[B21] Stenberg K, Tengborg C, Galbe M, Zacchi G (1998). Optimisation of steam pretreatment of SO_2_-impregnated mixed softwoods for ethanol production. J Chem Tech Biotechnol.

[B22] Sluiter A, Hames B, Ruiz R, Scarlata C, Sluiter J, Tempelton D (2004). Determination of structural carbohydrates and lignin in biomass.

[B23] Ruiz R, Ehrman T (1996). Dilute acid hydrolysis procedure for determination of total sugars in the liquid fraction of process samples.

[B24] Alkasrawi M, Rudolf A, Lidén G (2006). Influence of strain and cultivation procedure on the performance of simultaneous saccharification and fermentation of steam pretreated spruce. Enzyme Microb Technol.

[B25] Taherzadeh MJ, Lidén G, Gustafsson L, Niklasson C (1996). The effects of pantothenate deficiency and acetate addition on anaerobic batch fermentation of glucose by *Saccharomyces cerevisiae*. Appl Microbiol Biotechnol.

[B26] Söderström J, Galbe M, Zacchi G (2005). Separate versus simultaneous saccharification and fermentation of two-step pretreated softwood for ethanol production. J Wood Chem Technol.

[B27] Özcan S, Dover J, Johnston M (1998). Glucose sensing and signaling by two glucose receptors in the yeast *Saccharomyces cerevisiae*. EMBO J.

[B28] Lagunas R, Dominguez C, Busturia A, Sáez M (1982). Mechanisms of appearance of the Pasteur effect in *Saccharomyces cerevisiae*: Inactivation of sugar transport systems. J Bacteriol.

[B29] Busturia A, Lagunas R (1986). Catabolite inactivation of the glucose transport system in *Saccharomyces cerevisiae*. J Gen Microbiol.

[B30] Alonso A, Kotyk A (1978). Apparent half-lives of sugar transport proteins in *Saccharomyces cerevisiae*. Folie Microbiol.

[B31] Rudolf A, Alkasrawi M, Zacchi G, Lidén G (2005). A comparison between batch and fed-batch simultaneous saccharification and fermentation of steam pretreated spruce. Enzyme Microb Technol.

[B32] Sonderegger M, Jeppsson M, Larsson C, Gorwa-Grauslund MF, Boles E, Olsson L, Spencer-Martins I, Hahn-Hägerdal B, Sauer U (2004). Fermentation performance of engineered and evolved xylose-fermenting *Saccharomyces cerevisiae *strains. Biotechnol Bioeng.

